# Factors influencing extrathyroidal extension of papillary thyroid cancer and evaluation of ultrasonography for its diagnosis: a retrospective analysis

**DOI:** 10.1038/s41598-023-45642-x

**Published:** 2023-10-26

**Authors:** Hui Wang, Shanshan Zhao, Jincao Yao, Xiuhua Yu, Dong Xu

**Affiliations:** 1 Department of Ultrasound, Joint Service Support Force 903 Hospital, Hangzhou, China; 2 Department of Ultrasound, Shaoxing People’s Hospital, Shaoxing, China; 3 Department of Ultrasound, The Cancer Hospital of the University of Chinese Academy of Sciences (Zhejiang Cancer Hospital); Institute of Basic Medicine and Cancer (IBMC), Chinese Academy of Sciences, Key Laboratory of Head & Neck Cancer Translational Research of Zhejiang Province, Hangzhou, China

**Keywords:** Cancer, Oncology

## Abstract

Pathologists usually explore extrathyroidal extensions (ETEs) in thyroid cancer; however, sonographers are often not concerned with ETEs. We investigated factors influencing ETEs and the efficacy of ultrasound evaluation of thyroid capsule invasion. We retrospectively analysed 1933 papillary thyroid carcinoma patients who underwent thyroidectomy during 2018–2021. Patients were divided into three groups: no ETE, minor ETE (mETE), and gross ETE. Clinical characteristic differences were assessed using binary logistic regression analysis to identify ETE predictors, and the kappa test was performed to analyse consistency between ultrasonographic and pathological diagnoses of ETE. The mETE group was more likely to have larger tumour diameters and more extensive lymph node metastasis (LNM) than the no ETE group and more likely to be diagnosed in the isthmus. In the multivariate logistic regression analysis, longest tumour diameter, lesion site, LNM extent, and thyroglobulin concentration were significant mETE predictors. Minimal consistency existed between pathological and ultrasonographic examinations for neighbouring tissue invasion. Many clinical differences were observed between the no ETE and mETE groups, suggesting the importance of considering mETE. Therefore, sonographers should pay more attention to relationships between nodules and capsule and indicate these on ultrasound reports to provide more accurate preoperative ETE information for surgeons.

## Introduction

Papillary thyroid cancer (PTC) is the most common endocrine malignancy, and its incidence is increasing globally^1,2^. It is a major subtype of thyroid carcinomas and has a very low disease-specific mortality rate^3,4^. It is characterised by low invasiveness, high differentiation, and favourable response to treatment. However, extrathyroidal extension (ETE) is considered a risk factor for tumour recurrence and poorer prognosis^5–9^. Minor ETE (mETE) is a special type of ETE that has become a topic of significant research interest because its impact on tumours remains controversial^1,9–12^. The 2015 American Thyroid Association guidelines suggest that patients with mETE have a moderate risk of recurrence^6^ and therefore should receive a more active treatment. However, the eighth edition of the American Joint Commission on Cancer (AJCC) staging manual, which was implemented on 1 January 2018, states that gross ETE (gETE), identified either preoperatively or intraoperatively, is an important factor for staging. In contrast, mETE, whose analysis is limited to histologic examination of the thyroid capsule, is not included during staging evaluation^13,14^. The reasons are as follows: first, the subjective judgement of pathologists concerning the diagnosis of mETE can be diverse^15^. Second, many studies have reported that thyroid cancer invasion of the capsule has no significant effect on long-term survival^16–18^. However, pathological reports still describe in detail the invasion of the capsule fibres, fat, lymph nodes, extra-capsular fat, and/or the surrounding tissues of the capsule to indicate that the tumour cells have broken through the thyroid capsule without violating the banded muscle group. The significance of capsular and surrounding soft tissue invasion in thyroid cancer remains controversial.

Ultrasound (US) is the best imaging modality for evaluating thyroid nodules^19,20^ because it assesses the relationship between thyroid nodules and capsule, allowing professionals to judge whether ETE is present. Although several studies have suggested that US examination has favourable accuracy in diagnosing ETE^7,20–22^, in actual US reports, thyroid capsule invasion is often ignored or abbreviated, and a description of the relationship between specific nodules and capsule is often omitted. Some US evaluation reports only describe breaking through the capsule and not the proximity to the capsule. Thus, the question arises whether it is important for medical professionals who perform imaging to describe the relationship between the thyroid nodules and capsule in their reports. Therefore, in this study, the clinical data of patients with PTC were retrospectively analysed to explore differences among no ETE, mETE, and gETE and examine the diagnostic value of preoperative US examination for the detection of ETE.

## Results

### Clinicopathological characteristics of patients with PTC

Among 1,933 patients, 798 had ETE, 986 had mETE, and 149 had gETE; 1,116 patients had no central lymph node metastasis (LNM) and 817 had central LNM; and 1,642 patients had no lateral LNM and 291 had lateral LNM. Binary logistic regression was used to analyse the correlation between ETE and LNM. All *P* values were < 0.01, indicating that ETE was significantly correlated with central and lateral LNM. Clinical characteristics, including age, sex, longest tumour diameter, lesion site, central and lateral LNM, and TT3, TT4, fT3, fT4, TSH, TG-Ab, TPO-Ab, and Tg levels, were compared among the three groups. The results are shown in Table 1.

**Table 1:** Clinicopathological characteristics of 1933 patients with papillary thyroid carcinoma.

Variable	Extent of ETE			*P* value
	No ETE (n = 798, 41%)	mETE (n = 986, 51%)	gETE (n = 149, 8%)	
Age at diagnosis (years)^a^	43 (11–81)	46 (14–85)	47 (16–83)	
Age at diagnosis^b^				
< 55 years	608 (76)	736 (75)	112 (75)	0.752
≥ 55 years	190 (24)	250 (25)	37 (25)	
Sex^b^				
Male	209 (26)	271 (27)	41 (28)	0.818
Female	589 (74)	715 (73)	108 (72)	
Longest diameter^b^				
≤ 2 cm	760 (95)	856 (87)	100 (67)	< 0.001
> 2 cm	38 (5)	130 (13)	49 (33)	
Lesion site^b,c^				
Left	361 (45)	435 (44)	62 (42)	0.050
Right	418 (52)	500 (51)	79 (53)	
Isthmus	19 (2)	50 (5)	8 (5)	
Central lymph node metastasis^b^				
Yes	240 (30)	479 (49)	98 (66)	< 0.001
No	558 (70)	507 (51)	51 (34)	
Lateral lymph node metastasis^b^				
Yes	46 (6)	183 (19)	62 (42)	< 0.001
No	752 (94)	803 (81)	87 (58)	
Serum markers				
TT3 (ng/mL)^a^	1.07 (0.57–2.16)	1.07 (0.46–3.11)	1.07 (0.48–2.38)	0.977
TT4 (μg/dL)^a^	7.80 (1.32–14.80)	7.80 (3.60–14.00)	7.60 (1.30–15.90)	0.576
fT3 (pg/mL)^a^	3.21 (1.84–7.08)	3.20 (1.86–6.44)	3.20 (1.64–5.24)	0.494
fT4 (ng/dL)^a^	1.25 (0.62–2.75)	1.23 (0.66–2.56)	1.26 (0.31–2.76)	0.380
TSH (μIU/mL)^a^	1.504 (0.000–14.306)	1.624 (0.000–17.114)	1.711 (0.008–104.052)	0.028
TG-Ab (IU/mL)^a^	16.10 (9.85–3579.00)	15.55 (9.85–5539.35)	14.60 (9.85–618.70)	0.008
TPO-Ab (IU/mL)^a^	38.2 (4.15–1821.5)	34.8 (4.15–1821.5)	36.4 (4.15–1821.5)	< 0.001
Tg (ng/mL)^a^	11.000 (0.000–710.675)	12.925 (0.000–710.675)	20.430 (0.000–710.675)	< 0.001

*ETE*, extrathyroidal extension; *mETE*, minor extrathyroidal extension; *gETE*, gross extrathyroidal extension; *TT3*, triiodothyronine; *TT4*, thyroxine; *fT3*, free triiodothyronine; *fT4*, free thyroxine; *TSH*, thyroid-stimulating hormone; *TG-Ab*, anti-thyroglobulin; *TPO-Ab*, anti-thyroid peroxidase; *Tg*, thyroglobulin.

^a^Median and interquartile range, calculated using the multiple sample rank-sum test.

^b^Data are presented as n (%).

^c^One patient with papillary thyroid carcinoma had the entire thyroid affected.

There were no significant differences in age, sex, and TT3, TT4, fT3, and fT4 levels among the three groups. The longest tumour diameter, central and lateral LNM and TSH, TG-Ab, TPO-Ab, and Tg levels differed significantly among the groups. The lesion site was near-significant at unadjusted *P* value of 0.05, thus requiring further examination. The comparison of inconclusively identical features with *P* values of < 0.01 was repeated. These results are shown in Tables 2 and 3.

**Table 2:** Comparison of clinicopathological characteristics between the no ETE and mETE groups.

Variable	Extent of ETE		*P* value	χ^2^	Z
	No ETE	mETE			
Longest diameter					
≤ 2 cm	760	856	< 0.001	36.680	
> 2 cm	38	130			
Lesion site^a^					
Left	361	435	0.013	8.614	
Right	418	500			
Isthmus	19	50			
Central lymph node metastasis					
Yes	240	479	< 0.001	62.773	
No	558	507			
Lateral lymph node metastasis					
Yes	46	183	< 0.001	64.538	
No	752	803			
Serum markers					
TSH (μIU/mL)^b^	1.504	1.624	0.044		− 2.016
TG-Ab (IU/mL)^b^	16.1	15.55	0.023		− 2.271
TPO-Ab (IU/mL)^b^	38.2	34.8	< 0.001		− 3.715
Tg (ng/mL)^b^	11.000	12.925	< 0.001		− 3.578

*ETE,* extrathyroidal extension; *mETE,* minor extrathyroidal extension; *TSH,* thyroid-stimulating hormone; *TG-Ab,* anti-thyroglobulin; *TPO-Ab,* anti-thyroid peroxidase; *Tg,* thyroglobulin.

^a^One patient with papillary thyroid carcinoma had the entire thyroid affected.

^b^Median, assessed using the rank-sum test.

**Table 3:** Comparison of clinicopathological characteristics between the mETE and gETE groups.

Variable	Extent of ETE		*P* value	χ^2^	Z
	mETE	gETE			
Longest diameter					
≤ 2 cm	856	100	< 0.001	37.822	
> 2 cm	130	49			
Lesion site^a^					
Left	435	62	0.842	0.343	
Right	500	79			
I sthmus	50	8			
Central lymph node metastasis					
Yes	479	98	< 0.001	15.307	
No	507	51			
Lateral lymph node metastasis					
Yes	183	62	< 0.001	40.633	
No	803	87			
Serum markers					
TSH (μIU/mL)^b^	1.624	1.711	0.217		− 1.235
TG-Ab (IU/mL)^b^	15.55	14.6	0.126		− 1.531
TPO-Ab (IU/mL)^b^	34.8	36.4	0.643		− 0.464
Tg (ng/mL)^b^	12.925	20.43	< 0.001		− 4.477

*mETE*, minor extrathyroidal extension; *gETE*, gross extrathyroidal extension; *TSH*, thyroid-stimulating hormone; *TG-Ab*, anti-thyroglobulin; *TPO-Ab*, anti-thyroid peroxidase; *Tg*, thyroglobulin.

^a^One patient with papillary thyroid carcinoma had an entire thyroid affected.

^b^Median, assessed using the multiple sample rank-sum test.

There were significant differences in the longest diameter, tumour lesion site, LNM in the central region and lateral neck, and TSH, TG-Ab, TPO-Ab, and Tg levels between the no ETE and mETE groups (*P* < 0.05). The pairwise comparison of the three lesion sites showed significant differences between a left-sided position and the isthmus (*P* = 0.004) and a right-sided position and the isthmus (*P* = 0.004), although the difference between the left and right sides was non-significant (*P* = 0.940). There were significant differences in the longest diameter, LNM in the central region and lateral neck, and Tg levels between the mETE and gETE groups (*P* > 0.05).

### Related factors for neighbouring tissue invasion in patients in the no ETE and mETE groups

Since the eighth edition of AJCC no longer uses mETE as a staging standard, pathologists have found that the criteria for classifying patients into the no ETE group and mETE group are inconsistent with this adjustment. Therefore, we examined whether there are independent risk factors for neighbouring tissue invasion in the no ETE group compared with the mETE group. Binary logistic regression was used to analyse the factors influencing thyroid capsule soft tissue invasion. The results are shown in Table 4. Univariate and multivariate analyses identified the longest diameter, lesion site, central and lateral LNM, and preoperative Tg levels as independent factors of thyroid capsule soft tissue invasion.

**Table 4:** Factors associated with neighbouring tissue invasion in patients in the no ETE and mETE groups.

Variable	Univariate analysis			Multivariate analysis		
	OR	95% CI	*P* value	OR	95% CI	*P* value
Longest diameter^a^	1.102	1.083–1.123	< 0.001	1.093	1.071–1.116	< 0.001
Lesion site						
Not Isthmus	Ref			Ref		
Isthmus	1.299	1.086–1.553	0.004	1.292	1.074–1.555	0.007
Central lymph node metastasis						
Yes	Ref			Ref		
No	2.197	1.805–2.673	< 0.001	1.393	1.118–1.735	0.003
Lateral lymph node metastasis						
Yes	Ref			Ref		
No	3.726	2.656–5.225	< 0.001	2.046	1.394–3.001	< 0.001
Serum markers						
TSH (μIU/mL)^a^	1.091	1.009–1.180	0.028	1.083	0.998–1.175	0.057
TG-Ab (IU/mL)^a^	1.000	1.000–1.000	0.853			
TPO-Ab (IU/mL)^a^	1.000	1.000–1.000	0.215			
Tg (ng/mL)^a^	1.002	1.001–1.003	0.006	0.997	0.996–0.999	< 0.001

*OR*, odds ratio; *CI*, confidence interval; *Ref*, reference; *TSH*, thyroid-stimulating hormone; *TG-Ab*, anti-thyroglobulin; *TPO-Ab*, anti-thyroid peroxidase; *Tg*, thyroglobulin.

^a^Continuous variable.

**Figure 1:**
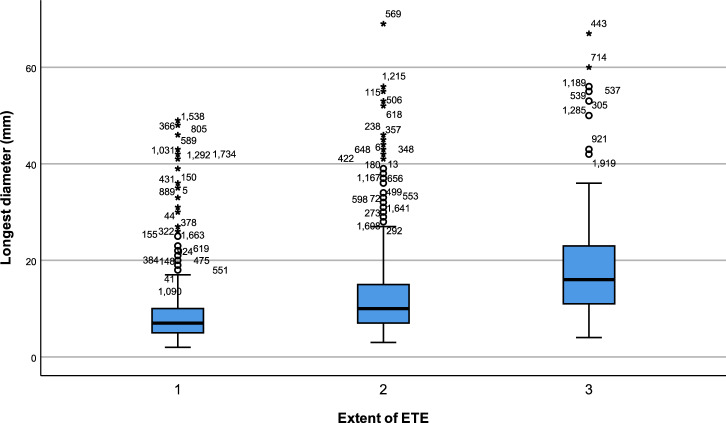
Box-and-whisker plot showing the distribution of longest tumour diameter in the three groups according to the extent of extrathyroidal extension (ETE) (*P* < 0.001). 1, Group 1 included patients with tumours confined to the thyroid. 2, Group 2 included patients with minor ETE. 3, Group 3 included patients with gross ETE.

The distribution of average tumour size, based on the extent of ETE, is presented in Fig. 1. The mean tumour diameters differed significantly among the ETE group (8.57 ± 6.25 mm), mETE group (12.63 ± 8.03 mm), and gETE group (18.67 ± 11.64 mm) (mETE vs no ETE, *P* < 0.001; gETE vs mETE, *P* < 0.001).

### Comparison of pathological and ultrasonic evaluation of ETE

Among 2,180 nodules, 958 (43.9%) had no ETE, 1,071 (49.1%) had mETE, and 151 (6.9%) had gETE. Ultrasonography showed that there were 2,053 nodules (94.2%) with no ETE, 122 (5.6%) with mETE, and 5 (0.2%) with gETE. There was almost no consistency between pathology and US results in terms of neighbouring tissue invasion (κ = 0.069, *P* < 0.01) (Table 5). Since we divided our patients into three groups based on ETE status, we could not evaluate sensitivity and specificity. With pathological results as the gold standard, the accuracy of US was 98.6% in the no ETE group. In contrast, for the mETE group, the accuracy of US was only 9.1%. For the gETE group, correct diagnosis was defined as the US evaluation reporting a breaking through of the capsule. The correct rate was only 10.6%, with an incorrect rate of 89.4%. Thus, the negative predictive value of US was much higher than the positive predictive value.

**Table 5:** Comparison of pathological and ultrasonic results of extrathyroidal extension (ETE).

Pathology	Ultrasonography			Sum	*Kappa*	*P* value
	1	2	3			
1	945	13	0	958	0.069	< 0.001
2	973	97	1	1071		
3	135	12	4	151		
Sum	2053	122	5	2180		

1, Group 1 included patients with tumours confined to the thyroid; 2, group 2 included patients with minor ETE; 3, group 3 included patients with gross ETE.

## Discussion

This study found that there were significant differences in the longest tumour diameter; lesion site; central and lateral LNM; and TSH, TG-Ab, TPO-Ab, and Tg levels between the no ETE and mETE groups (*P* < 0.05). However, only the longest tumour diameter, central and lateral LNM, and Tg levels differed significantly between the mETE and gETE groups (*P* < 0.05). Our analysis of the no ETE and mETE groups showed that the longest tumour diameter, lesion site, central and lateral LNM, and preoperative Tg levels were independent predictors of thyroid capsule invading soft tissue. Preoperative US findings of ETE was inconsistent with the pathology findings, a result that differs from previous studies^7,20–22^.

Most PTCs show a slow and inert growth pattern. The 5-year survival rate is currently > 90%^7,23^. However, a large proportion of patients with PTC experience recurrence. Due to different follow-up times, the recurrence rate ranges from 20 to 35%^7,24,25^. ETE of thyroid carcinoma, referring to the invasion of the primary tumour into the adjacent tissues beyond the gland, is a well-established risk factor for recurrence and adverse outcomes in patients with PTC^5,6^. The presence of ETE of a tumour affects the surgical approach and increases the risk of incomplete surgical excision, which in turn is associated with excess morbidity and increased mortality risk^21^. On the other hand, if the scope of surgery is expanded due to misdiagnosis of ETE, interventions are affected by more severe complications in more demolitive surgery^26^. Therefore, ETE is an important factor in determining the required surgical range^27^.

Recently, the AJCC concluded that mETE, detected only on histological examination, has an insignificant impact on mortality. They proposed that only gETE, which refers to the macroscopic invasion of a tumour on imaging or during surgery, is clinically relevant and, hence, impacts the staging of PTC. In the updated eighth version of the AJCC staging system, the presence of mETE no longer constitutes a T3 category, and gETE to the strap muscle is newly designated as the T3b category^14–16,28^.

The reasons for this change are as follows. First, there is no embryological, anatomical, or histological evidence that the thyroid gland has a continuous or homogeneous fibrous capsule. The connective tissue observed at the outer surface of the thyroid gland should be interpreted as a pseudocapsule composed of surrounding fibroadipose tissue originating from the lateral mesodermal plate^15,29^. Second, concordance among pathologists about the presence of ETE is higher when it is assessed using the relationship between carcinoma and skeletal muscle; however, low inter-observer agreement is noted when only adipose tissue, vasculature, and nerves are considered^8,15^. Third, several studies have failed to show a difference in recurrence or mortality rates between patients without ETE and patients with microscopic ETE^16–18,30,31^. However, this issue remains controversial. Some studies have shown that the recurrence rate of patients with ETE diagnosed by microscopy is higher than that of patients without ETE^1,9–12,32^. On the contrary, the negative impact of ETE invading major structures (i.e. T4 disease in the AJCC TNM staging system) on recurrence and mortality is widely accepted^5,7,8,21,25,33,34^. Therefore, ETE assessments should be carefully conducted to reduce the tumour recurrence rates and improve patient prognoses.

This retrospective study did not assess recurrence rates and patient prognoses; therefore, we could not conclusively determine the clinical meaningfulness of mETE in the context of PTC. However, although the AJCC eighth edition states that the presence of mETE is no longer regarded as a staging standard, our results show that pathologists still attach great importance to the state of the thyroid capsule. Therefore, with the attention of pathologists, the number of patients in the mETE group of this study accounted for 49.1% of the study cohort. Although we classified patients in this study into three groups, the focus was on the difference between the no ETE and mETE groups only. Even though the AJCC does not take thyroid capsule soft tissue invasion into account for TNM staging for the aforementioned reasons, we found many differences in the clinicopathological features between the no ETE and mETE groups. Furthermore, since ETE is also an independent risk factor for LNM, we suggest that the importance of mETE cannot be ignored.

Our findings suggest that a larger tumour size is a predictor of ETE, which is not only logical, but also consistent with the results of several previous studies^1,3,35^. Our study suggests that PTC in the isthmus or the left and right thyroid is also a predictor of ETE, which is consistent with the findings of at least one previous study^36^. Similar to tumour size, US evaluation and surgeon assessment are also largely based on tumour location. Both benign and malignant tumour US evaluation reports usually describe the tumour location in detail; thus, there is no concern of it losing focus. The relationship between ETE and LNM in the central region and the lateral neck is bidirectional. Several studies have shown that ETE is an independent predictor of LNM^9,20,34,37–41^. However, several retrospective studies have shown an association between mETE and the presence of LNM, indicating that mETE is a marker of disease biology in PTC^3,10,13^. The results of our study are consistent with those of these retrospective studies. Similarly, US and surgeon evaluations place great importance on LNM.

The final factor influencing ETE was preoperative serum Tg levels. Few studies assess Tg because it is susceptible to various factors^42^. Despite the large sample size of this study, Tg level was the only significant indicator among all assessed serum markers. Therefore, future studies should assess the viability of preoperative Tg level as a diagnostic indicator of ETE in patients with PTC.

In terms of ETE, US can identify the relationship between the thyroid capsule and the anterior cervical muscle group^7^. Ideally, the radiologic impression should be corroborated by the surgeon’s intraoperative findings and/or the pathologist’s assessment for ETE^43^. Numerous sonographic features have been previously associated with ETE with varying importance: irregular or indistinct margins, capsular abutment, subtle capsular distortion, long interface of the thyroid capsule with the nodule, and/or the presence of a tracheal footprint^1,16,43,44^. The degree of contact between the nodule and the capsule, the bulging of the thyroid capsule contour, and echo disruption, among other indicators, are valuable for predicting extra-capsular invasion, despite being subjective and reliant on the visual judgement of the examiner^7,21^. Thus, although no single sonographic feature was highly predictive of ETE, careful evaluation of the capsule combined with the examination of sonographic features may help in biopsy selection, surgical planning, and treatment of patients with PTC^21^.

**Figure 2:**
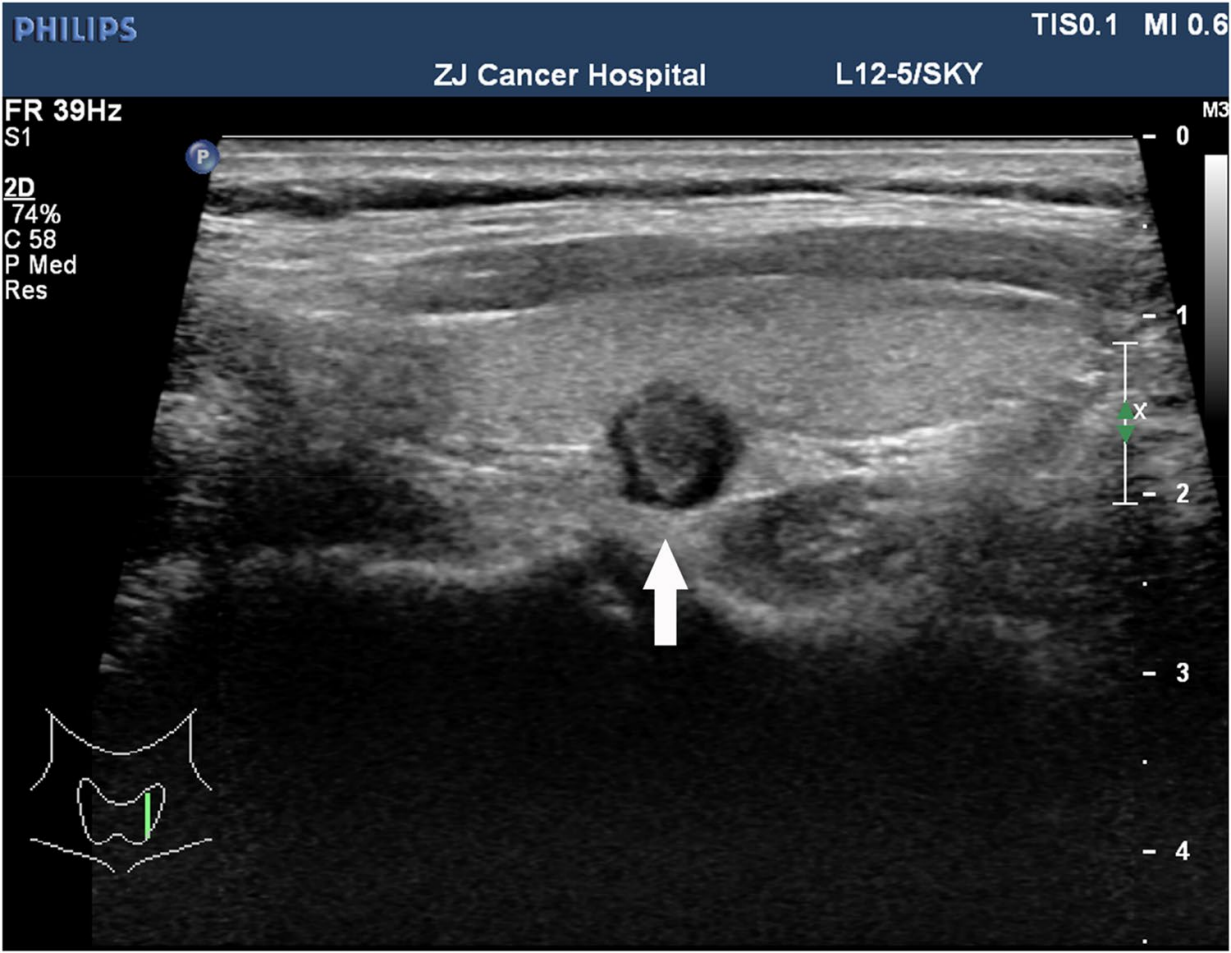
An ultrasound image of a 51-year-old female patient with papillary thyroid carcinoma.

**Figure 3:**
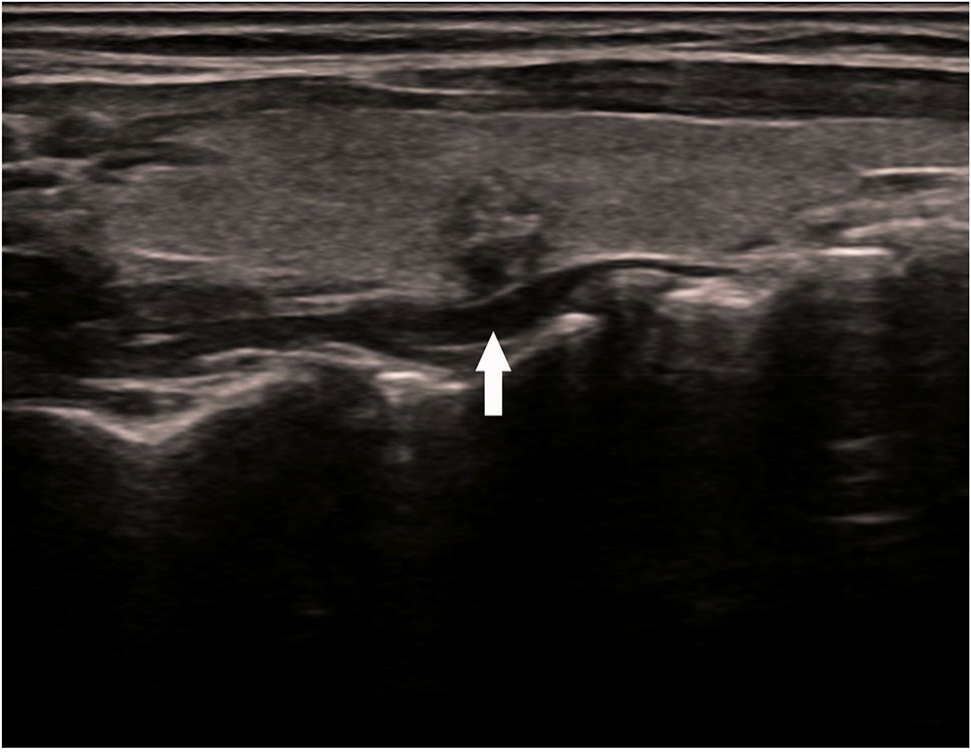
An ultrasound image of a 41-year-old female patient with papillary thyroid carcinoma.

The preoperative US findings of ETE in this study were not consistent with the pathology results. The reasons for this were hypothesised as follows: first, in the eighth edition of AJCC, T3b was changed to banded muscle invasion, and the concept of minimum capsule invasion was excluded. As a result, US evaluations no longer report on the relationship between nodules and the capsule. Second, there are no standard ultrasonic characteristics for evaluating invasion beyond the tumour capsule boundary, leading to reliance on personal experience. Third, because there is no uniform standard, many US evaluation reports subjectively ignore the relationship between nodules and the capsule. For example, even if obvious nodules break through the capsule, US reports do not mention this aspect. Figure 2 is an ultrasound image of a 51-year-old female patient with PTC. The associated pathological report showed PTC, focal interstitial collagenisation and calcification, recurrent capsule and surrounding striated muscle tissue carcinoma. Figure 2 shows that the continuity of the posterior capsule of the thyroid was interrupted by the nodules breaking through. However, the US evaluation report did not suggest ETE. Figure 3 is an ultrasound image of a 41-year-old female PTC patient. The pathological report showed papillary thyroid microcarcinoma with interstitial collagenisation and calcification. The US shows that the boundary between the nodules and the capsule was unclear, which was consistent with mETE imaging characteristics, but the pathological report did not indicate mETE. This shows that correct diagnosis via US was more difficult in critical cases.

Our study suggests that US reports underestimate whether the description of the capsule meets the latest criteria because the accuracy of US reports describing the pathological characteristics is low in not only mETE but also gETE. Some nodules with obvious breakthrough in the capsule did not appear in the US report, indicating that the failure to report this may affect the surgeon’s preoperative judgement of thyroid tumours. Although numerous studies have proposed many ultrasonic features of neighbouring tissue invasion, it is nearly impossible for US doctors to write detailed features in their reports during practical work. However, we recommend indicating ‘whether invasion of the neighbouring tissue has occurred’ in US reports since surgeons are more concerned about the contents of reports than the US images. Building on the findings of existing studies, our study suggests that the contact degree between nodules and capsule ≥ 25%, the obvious protrusion of the thyroid capsule contour, and the obvious interruption of echo can be used as criteria to identify ETE during US evaluation. If the contact degree between nodules and capsule is < 25%, the thyroid capsule contour is slightly protruding, and the echo is suspiciously interrupted, the US report can describe ‘nodules close to the capsule’ or ‘suspected ETE’, which is sufficient for surgeons. Our study suggests that US doctors should pay attention to the relationship between nodules and capsule in PTC patients and describe it in detail as much as possible in the US report. Then, the surgeon can determine the scope of surgery based on such an US report, thus ensuring that patients receive better treatment and potentially improved prognosis.

This study has some limitations. First, our results cannot be generalised as all patients included in this study were from China. Second, the results of our study are from the same hospital; therefore, they are not necessarily reflective of cases at other hospitals. Third, due to the large sample size, different brands of ultrasonic instruments were used, resulting in the image presentations being inconsistent. Fourth, since this is a retrospective study, the ultrasound examiners were not specifically instructed to look at ETE. Fifth, the US analysis might have been affected by subjective factors, such as the thyroid capsule contour slightly protruding or the echo being suspiciously interrupted. Whether ETE is included in the US report is also related to the habits of reporting personnel, and the results of the report may deviate from the actual image. Lastly, there are no standardised criteria for assessing ultrasonic characteristics and diagnosing ETE.

In conclusion, there were some differences in clinical characteristics between the mETE and no ETE groups as well as between the mETE and gETE groups. The study findings suggest that preoperative US reports should not ignore the relationship between nodules and the thyroid capsule. Preoperative US evaluation of ETE was inconsistent with the pathology findings. The lack of an ETE preoperative prompt for surgeons was linked to the difficulty in objective evaluation and deliberate disregard of ETE in US evaluation reports. US evaluation personnel should therefore carefully assess the relationship between nodules and the thyroid capsule. Furthermore, this assessment should be included in the US reports to facilitate improved surgical guidance on ETE identification and associated decisions.

## Materials and methods

### Patients

This retrospective study included 1,933 patients with PTC recruited from the Cancer Hospital of the University of Chinese Academy of Sciences (Zhejiang Cancer Hospital) who underwent thyroidectomy from August 2018 to February 2021. We included patients with PTC who underwent total or hemi-thyroidectomy and had complete clinical data. Patients were excluded from the study based on the following criteria: (1) underwent non-curative surgery; (2) underwent reoperation; (3) had non-PTC pathological type of thyroid cancer; (4) did not undergo testing for preoperative serological indicators; or (5) had incomplete clinical data or missing follow-up data. Finally, 1933 patients (521 men and 1412 women) with 2180 lesions were enrolled in this study. The patients were aged between 11 and 85 years (mean: 45.14 ± 12.24 years). The Medical Ethics Committee of Zhejiang Cancer Hospital approved this study (approval number: IRB-2020-287) and waived the requirement of obtaining informed consent. All procedures performed in this study involving human participants were in accordance with the Declaration of Helsinki (as revised in 2013).

### Clinicopathological data

ETE was subclassified based on previous studies^15,27^: mETE, a tumour extending beyond the thyroid capsule into the surrounding peri-thyroidal soft tissues of fat and/or capsule fibre; and gETE, visual evidence of tumour invasion into the strap muscles, subcutaneous soft tissue, larynx, trachea, oesophagus, recurrent laryngeal nerve, and/or prevertebral fascia. Patients and nodules were divided into three groups according to the findings of the pathological reports. Group 1 included patients with tumours confined to the thyroid (no ETE), group 2 included patients with mETE, and group 3 included patients with gETE. Differences in the clinical characteristics of the three groups were assessed.

### Ultrasonic instruments and inspection methods

The US procedures were performed using GE Logiq E9 (General Electric (GE) Healthcare, Chicago, IL, USA), Toshiba APLIO 400 TUS-A400 (Toshiba Medical Systems, Otawara, Japan), and Hitachi Arietta 70 (Hitachi, Tokyo, Japan) colour Doppler US detectors at a probe frequency of 5–15 MHz. All patients underwent colour Doppler ultrasonography before surgery, which was performed by three diagnosticians with more than three years of thyroid imaging experience. The chief physician was consulted on cases requiring additional assessment prior to final diagnostic determination. Patients were placed in the supine position, with their neck fully exposed. The lesions observed on US were carefully evaluated for diagnostic features, including size, location, shape, calcification, and boundary. The cervical lymph nodes were also assessed carefully. For each thyroid nodule, the tumour diameters were measured on the antero-posterior, transverse, and longitudinal planes. Tumour size was defined using the greatest diameter measurement for each lesion. ETE interpretations were inconsistent because diagnosticians performing US examination had a varying understanding of ETE. Moreover, the eighth edition of AJCC was implemented in 2018. Thus, there was no uniform standard for the ultrasonic characteristics for ETE diagnosis. The study sonographer characterised ETE as an obvious capsular base, protruding contour, and disappearance of the thyroid capsular echo. Based on the ultrasound report findings, a description of ‘close to the capsule’ was defined as ultrasound mETE and ‘invasion of banded muscle’ as ultrasound gETE.

### Detection of serum markers

Triiodothyronine (TT3, reference range: 0.66–1.92 ng/mL), thyroxine (TT4, reference range: 4.3–12.5 μg/dL), free triiodothyronine (fT3, reference range: 1.80–4.10 pg/mL), free thyroxine (fT4, reference range: 0.81–1.89 ng/dL), thyroid-stimulating hormone (TSH, reference range: 0.380–4.340 μIU/mL), anti-thyroglobulin (Tg-Ab, reference range: 0.0–60.0 IU/mL), and anti-thyroid peroxidase (TPO-Ab, reference range: 0.0–60.0 IU/mL) levels were measured before surgery using an ADVIA Centaur automatic chemiluminescence analyser (Siemens, Munich, Germany). Fasting serum thyroglobulin (Tg, reference range: 1.40–78 ng/mL) levels were measured using Cobase 8000 E602 (Roche Diagnostics, Mannheim, Germany). If the test value exceeded the detection range of the instrument, group spacing was estimated using the interval grouping method, and the value was replaced.

### Surgical treatment

At our institution, prophylactic central lymph node dissection was performed in all patients with preoperative imaging examination indicating thyroid cancer. Total thyroidectomy was performed in patients identified with ETE, multi-focality, or LNM during preoperative or intraoperative examination. Lateral compartment lymph node dissection was performed only in patients with suspicious LNM on US or with confirmed lateral neck nodal metastases based on preoperative fine-needle aspiration biopsy.

### Statistical analyses

SPSS version 25.0 statistical software (IBM Corporation, Armonk, NY, USA) was used to analyse the data. The chi-square test was used to compare categorical variables among the groups. The measurement data were tested for normality using the Shapiro–Wilk and Kolmogorov–Smirnov tests. The data with non-normal distribution are expressed as medians and ranges. The Wilcoxon rank-sum test was used to compare the differences in each measurement variable between the three groups. Factors with a *P* value < 0.05 were assessed using multivariable logistic regression analysis. The independent related factors of no ETE and mETE group were screened by forward stepwise screening method based on likelihood ratio test (*P* < 0.05). Odds ratios (ORs) and 95% confidence intervals (CIs) were calculated. The count data are expressed as cases, and agreement between the consistency of the US examinations and the pathological findings was determined using the kappa statistic. Kappa values between 0.21 and 0.40 indicate low consistency; between 0.41 and 0.60, medium consistency; and between 0.61 and 0.80, high consistency.

## Data Availability

The datasets generated analysed during the current study are available from the corresponding author on reasonable request.
